# Effect of obesity on the effectiveness of cardiac resynchronization to reduce the risk of first and recurrent ventricular tachyarrhythmia events

**DOI:** 10.1186/s12933-016-0401-x

**Published:** 2016-07-07

**Authors:** Barbara Szepietowska, Bronislava Polonsky, Saadia Sherazi, Yitschak Biton, Valentina Kutyifa, Scott McNitt, Mehmet Aktas, Arthur J. Moss, Wojciech Zareba

**Affiliations:** Heart Research Follow-up Program, Cardiology Division, University of Rochester Medical Center, 265 Crittenden Blvd., PO Box 653, Rochester, NY 14642 USA

**Keywords:** Obesity, Cardiac resynchronization therapy, Implantable cardioverter defibrillator, Heart failure, Ventricular tachyarrhythmias

## Abstract

**Background:**

Obesity is associated with multiple adverse cardiovascular conditions and may increase the risk of ventricular tachyarrhythmias (VT/VF). There is limited data on the association between obesity and risk of VT/VF requiring appropriate implantable cardioverter-defibrillator (ICD) therapies and the effectiveness of cardiac resynchronization therapy (CRT) to reduce risk for VT/VF. The multicenter automatic defibrillator implantation trial with cardiac resynchronization therapy (MADIT-CRT) was design to investigate effectiveness of CRT therapy to reduce cardiovascular outcome for patients with heart failure (HF) and reduced ejection fraction.

**Methods and results:**

We identified patients enrolled in the MADIT CRT trial as obese (n = 433) and non-obese (n = 845) and analyzed their risk for appropriate device therapy for VT/VF, repeated VT/VF events, fast VT/VF, as well as events after first VT/VF episodes. Obesity was defined as body mass index (BMI) ≥30 kg/m^2^. Among ICD patients, the risk of first appropriate ICD therapy for VT/VF at 3 years was similar between obese and non-obese patients (23 vs. 21 %, p = 0.76). CRT-D treatment reduced the risk of first appropriate ICD therapy both in non-obese ([HR]; 0.58 [CI]: 0.42–0.79; p < 0.001) and obese patients (HR 0.75, 95 % CI 0.5–1.38; p = 0.179) (interaction p value 0.323). Similarly, a significant reduction in the risk of fast VT/VF was observed in non-obese patients ([HR]; 0.49 [CI]: 0.33–0.73; p < 0.001) and obese ([HR]; 0.49 [CI]: 0.29–0.81; p < 0.01), (interaction p value 0.984).

**Conclusion:**

Obese and non-obese patients with mild heart failure have a similar risk of ventricular tachyarrhythmias. Obesity in mild heart failure did not diminish the clinical benefit of cardiac resynchronization therapy to reduce risk for appropriate ICD therapy.

*Clinical trial registration*http://clinicaltrials.gov/ct2/show/NCT00180271

**Electronic supplementary material:**

The online version of this article (doi:10.1186/s12933-016-0401-x) contains supplementary material, which is available to authorized users.

## Background

Obesity contributes to the development of several risk factors that are associated with cardiac arrhythmias, including metabolic syndrome, ischemic heart disease [1, 2], atrial enlargement, left ventricular hypertrophy [3], systolic and diastolic heart failure [4, 5] and sleep apnea [6]. The cardiac structural abnormalities that are associated with obesity may potentially increase the risk for ventricular arrhythmogenesis including myocyte hypertrophy, fibrosis, focal myocardial disarray, fatty infiltration and increased epicardial fat [7]. Patients with obesity have also been shown to have disrupted pattern of gap junction protein expression and distribution. [8] Obesity is also associated with delayed ventricular repolarization as evidenced by prolongation of QT/QTc interval [8, 9]. Additional mechanism responsible for increased risk for arrhythmias is sympathetic over activation present in patients with obesity [10]. These changes in part may contribute to the increased propensity to VT/VF in obese individuals [11].

The association between atrial fibrillation and obesity has been thoroughly investigated [12–16], however the association between ventricular arrhythmias and obesity is not fully understood. Increased prevalence for cardiac tachyarrhythmias and increased risk of sudden cardiac death was reported in obese post myocardial infarction patients with abnormal ejection fraction [17–19].

In our previous MADIT II, where ICD therapy was tested for prevention for sudden cardiac death, we observed that obesity is an independent risk factor for VT/VF [17]. The aim of the current study was to: (i) evaluate the association between obesity and the risk of appropriate implantable cardioverter-defibrillator (ICD) therapy delivered for VT/VF in patients with mild heart failure and reduced ejection fraction; (ii) evaluate the effectiveness of cardiac resynchronization therapies to reduce risk for VT/VF in obese and non-obese patients, (iii) to assess the prognostic implications of first VT/VF on the subsequent tachyarrhythmia event and all-cause mortality in this population.

## Methods

### Study population

The results and the protocol of the Multicenter Automatic Defibrillator Implantation Trial with Cardiac Resynchronization Therapy (MADIT-CRT) trial have been previously reported [20]. From December 22nd, 2004, through June 24th, 2009 a total of 1820 patients were enrolled at 110 centers in US, Canada and in Europe. Patients of either sex who were at least 21 years old, with ischemic cardiomyopathy (NYHA class I or II) or non-ischemic cardiomyopathy (NYHA class II only), sinus rhythm, a left ventricular ejection fraction (LVEF) of 30 % or less and prolonged intraventricular conduction (QRS duration ≥130 ms) were randomly assigned in 3:2 ratio to CRT-D or ICD only. Patients had an ambulatory follow-up one-month after the device implantation, and every 3 months thereafter until the termination of the trial. The mean follow-up of the enrolled patients was 40 months. All patients had clinical evaluation at each follow up visit or at any meaningful clinical event.

Following the primary publication of MADIT-CRT, subsequent analyses showed that the benefit of CRT-D in the trial was restricted to patients with left bundle branch block (LBBB) [21]. Patients with obesity were defined as BMI ≥30 kg/m^2^ at the baseline visit [22].

### Device programming and Interrogation

Commercially available transvenous devices from Boston Scientific and standard techniques were used in the MADIT-CRT. Devices were programmed according to the study protocol [23] to monitor therapy, with a protocol recommendation to a setting of the VT zone at 180 beats/min (bpm) and the VF zone at 210 bpm. Sensitivity was programmed according to physician discretion. Detection times were 2.5 s for the VT zone and 1.0 s for the VF zone. The protocol recommended programming the VT zone with first therapy to burst-type antitachycardia pacing with 8 pulses at 88 % of the measured cycle length with a 10-milliseconds decrement between bursts, then shock therapy; second therapy was recommended to be shock at the defibrillation threshold plus at least 10 J (if possible). The remaining therapies were to be maximal energy shocks. All shocks were biphasic. The ICDs were interrogated quarterly, after which ICD data and disks were sent to the core laboratory for categorization and final adjudication of detected arrhythmias. Arrhythmia episode was defined as any type of therapy rendered including antitachycardia pacing and shock. The adjudication committee adjudicated the episode. VT was defined as the ventricular rate up to 250 bpm; VF was defined as ventricular rate faster than 250 bpm with disorganized ventricular electrographs. Only appropriate ICD therapy delivered for VT (≥180 bpm) or VF was considered in the present study.

### End points definition

The end point of the current study was first VT/VF requiring appropriate ICD therapy, repeated VT/VF requiring appropriate ICD therapy, VT/VF or death whichever came first, fast VT/VF defined as >200 bpm and VT/VF requiring ICD shocks.

### Statistical analysis

Baseline clinical characteristics were compared using the nonparametric Kruskal–Wallis test for continuous variables and the Chi square-test or Fisher’s exact test for dichotomous variables, as appropriate. We performed Kaplan–Meier survival analyses of unadjusted cumulative event rates stratified by obesity with the log-rank test for determination of statistical significance.

The Cox proportional hazards multivariate regression model was used to estimate hazard ratios for risk of appropriate ICD therapy delivered for (VT/VF), VT/VF/death, VT/VF greater than 200 bpm and shock delivered for VT/VF. These hazard ratios were estimated for two separate groups: non-obese and obese patients. The independent variables were chosen using the best subsets selection method and adjustment included: assigned treatment, race (Black/African American), age at enrollment, creatinine ≥1.4, female, left ventricle end diastolic volume index, myocardial infarction prior to enrollment, enrollment NYHA classification, prior hospitalization during preceding year, QRS <150 ms, ventricular arrhythmias requiring treatment prior to enrolment. The full multivariable model is presented in Additional file 1: Table S1. We followed this statistical methodology because we wanted to develop a parsimonious model which excluded variables which were not significantly predictive of the endpoints and would have very little impact on the results. In this way we attempted to maximize statistical power, an important consideration in subgroup analysis. To assess the CRT-D treatment differences between patients by obesity, a treatment-by-obesity medication interaction term was included in the Cox proportional hazard regression models. A two degree of freedom Wald test was done to assess the strength of the interaction between the groups and CRT-D treatment. All statistical tests were two-sided and a p < 0.05 was considered statistically significant, because of the numerous statistical tests, the p value reported should be considered as nominal and not corrected for multiple comparison. Analyses were carried out with SAS software (version 9.3, SAS institute, Cary, North Carolina).

## Results

MADIT CRT in total included 1820, 539 were excluded due to non-LBBB and 17 due to not having BMI at baseline. The study population consisted of 1264 patients with LBBB including 833 (66 %) non-obese and 431 (34 %) obese patients. Clinical and demographic characteristics of the study population are presented in Table 1. In summary, obese patients were younger by about 5 years, more often presented with diabetes and hypertension, and had longer QRS duration, higher resting heart rate, systolic and diastolic blood pressure, higher glomerular filtration rate (GFR) and lower plasma brain natriuretic peptide (BNP) () levels. Patients with obesity more often reported usage of diuretics including aldosterone receptor antagonists. ICD programming was also not different between non-obese and obese. A total of 266 (21 %) LBBB patients experienced at least one tachyarrhythmia event at a heart rate ≥180 beats/min.

**Table 1:** Clinical characteristics of LBBB patients by obesity in MADIT-CRT

	Non-obese n = 833	Obese n = 431	p value
Demographics			
Age, mean ± SD, y	65.8 ± 10.6	61.1 ± 10.6	<0.001
Women, n (%)	263 (32)	125 (29)	0.348
White n (%)	764 (92)	389 (90)	0.272
Cardiac history n (%)			
CRT-D assigned n (%)	498 (60)	259 (60)	0.915
Ischemic cardiomyopathy	374 (45)	183 (42)	0.408
Diabetes	216 (26)	166 (39)	<0.001
Hypertension	497 (60)	300 (70)	<0.001
Prior MI	268 (33)	135 (32)	0.761
Prior CABG	191 (23)	90 (21)	0.401
Prior HF hospitalization	310 (38)	172 (40)	0.421
Past atrial arrhythmias	93 (11)	47 (11)	0.892
Past ventricular arrhythmias	47 (6)	34 (8)	0.121
Clinical characteristics at enrolment mean ± SD			
LVEF (%)	28.9 ± 3.5	28.5 ± 3.4	0.132
QRS duration (ms)	162.1 ± 19.3	164.7 ± 19.0	0.012
Resting heart rate (bpm)	67.7 ± 10.9	69.2 ± 10.9	0.019
Systolic blood pressure (mmHg)	122.2 ± 17.2	123.6 ± 17.0	0.090
Diastolic blood pressure (mmHg)	70.7 ± 9.9	72.9 ± 10.7	0.002
Brain natriuretic peptide pg/dl	134.8 ± 168.9	78.7 ± 91.9	<0.001
Glomerular filtration rate ml/m^2^	68.5 ± 19.8	70.8 ± 20.4	0.046
Medications, n (%)			
ACE inhibitor or aldosterone receptor antagonists	797 (96)	418 (97)	0.254
Aldosterone receptor antagonists	254 (30)	172 (40)	<0.001
Aspirin	500 (60)	275 (64)	0.191
Beta-blockers	779 (94)	408 (95)	0.419
Diuretic	536 (64)	328 (76)	<0.001
Statins	522 (63)	281 (65)	0.375
ICD programming			
Rate of lowest VT <180 bpm	153 (20)	63 (16)	0.060
Rate of highest VF >210 bpm	79 (10)	45 (10)	0.650
ATP (%)	743 (91)	399 (93)	0.267
Cut-off rate of lowest VT zone (ms)	177 ± 7	178 ± 7	0.029
Cut-off rate of VF zone (ms)	209 ± 9	210 ± 8	0.357

### The appropriate ICD therapies in obese and non-obese patients

**Fig. 1:**
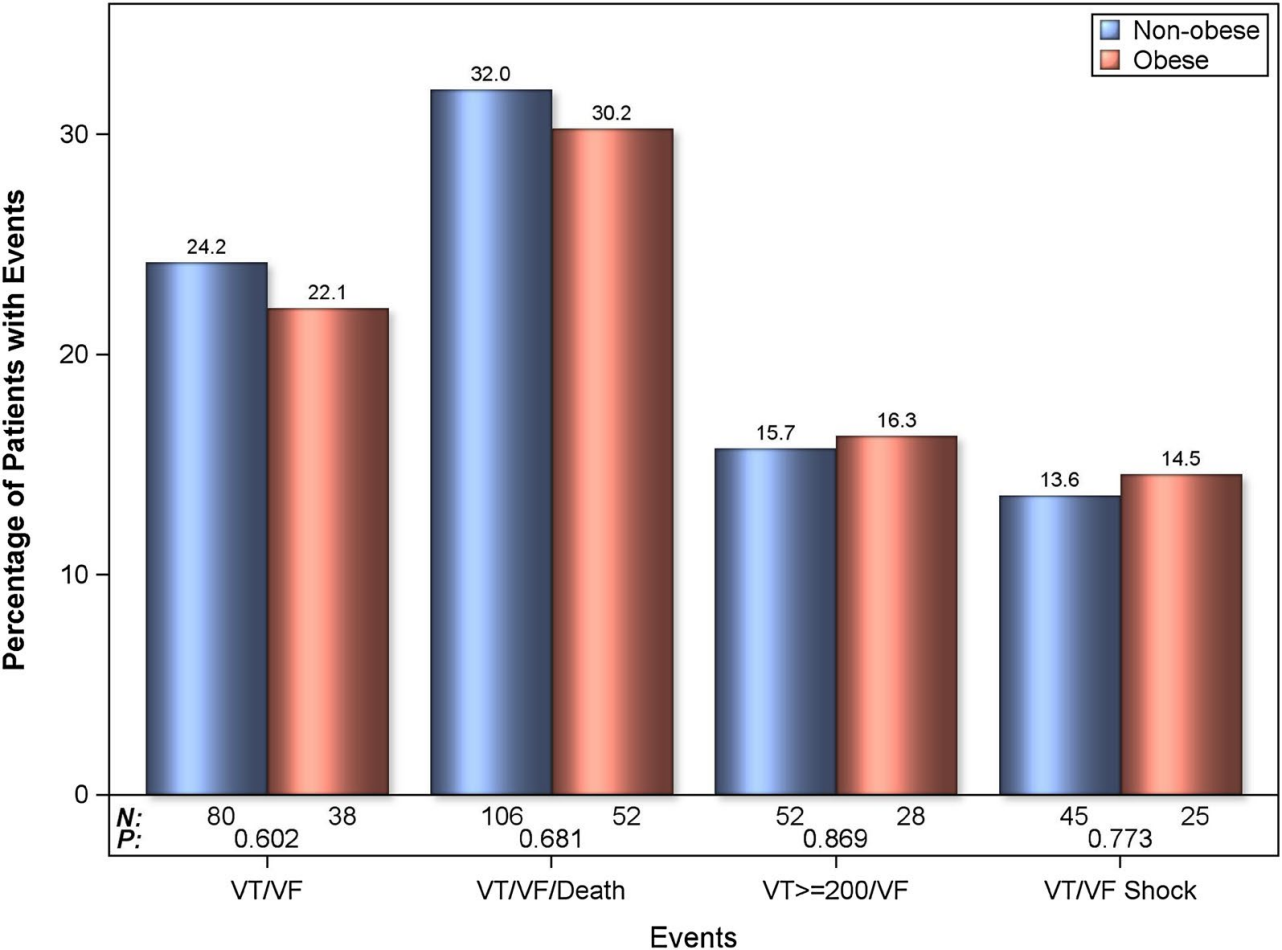
Percentage of patients with appropriate ICD therapy for VT/VF at 3 years in non-obese and obese in ICD arm

Among patients in the ICD arm only, the risk for appropriate ICD therapy at 3 years was similar between obese and non-obese patients for VT/VF, VF/VF/death, as well as shock delivered for VT/VF, and VT/VF higher than 200 (Fig. 1). Multivariable analysis consistently showed that risk for appropriate therapy in ICD arm was similar for non-obese and obese patients (Table 2).

**Table 2:** Risk of appropriate implantable cardioverter-defibrillator therapy in obese versus non-obese patients in ICD arm

	Number events	Adjusted HR (95 % CI) p value
VT/VF	124	1.33 (0.91–1.96) 0.144
VT/VF/death	159	1.25 (0.89–1.75) 0.199
VT/VF greater than 200 bpm	62	0.96 (0.57–1.64) 0.880
Shock delivered for VT/VF	76	0.96 (0.67–1.79) 0.745

After adjustment for: race (Black/African American), age at enrollment, creatinine ≥1.4, female sex, left ventricle end diastolic volume index, myocardial infarction prior to enrollment, enrollment NYHA classification, prior hospitalization during prior year, QRS <150, ventricular arrhythmias requiring treatment prior to enrolment

In our population non-obese and obese presented with similar rate for appropriate therapy in ICD arm, CRT-D arm and both arm combined (Table 3) and (Additional file 2: Figure S1).

**Table 3:** Rates of recurrent appropriate ICD therapies for VT/VF per 100 patient-years at risk assessed at a 3-year follow-up

Treatment arm	Events	Non-obese	Obese	p value
ICD	VT/VF	56.82	44.58	0.244
	VT/VF/Death	61.3	48.44	0.237
	VT/VF greater than 200 bpm	23.94	19.29	0.384
	Shock delivered for VT/VF	22.71	18.43	0.522
CRT-D	VT/VF	27.97	40.22	0.453
	VT/VF/death	30.95	42.85	0.359
	VT/VF greater than 200 bpm	9.25	7.49	0.495
	Shock delivered for VT/VF	8.72	5.41	0.171

### The effect of CRT-D on the risk of appropriate implantable cardioverter-defibrillator therapy

**Fig. 2:**
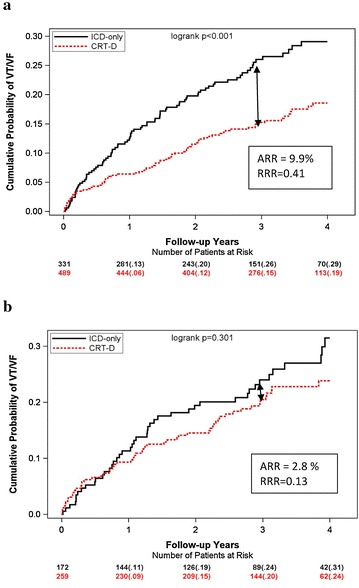
Cumulative probability of VT/VF by treatment arm in: **a** non-obese and **b** obese patients

Kaplan–Meier survival analysis showed the cumulative probability of the of first appropriate ICD therapies delivered for VT/VF was significantly decreased for non-obese patients obese did not demonstrate a decreased when CRT-D was compared to ICD group only (Fig. 2).

**Fig. 3:**
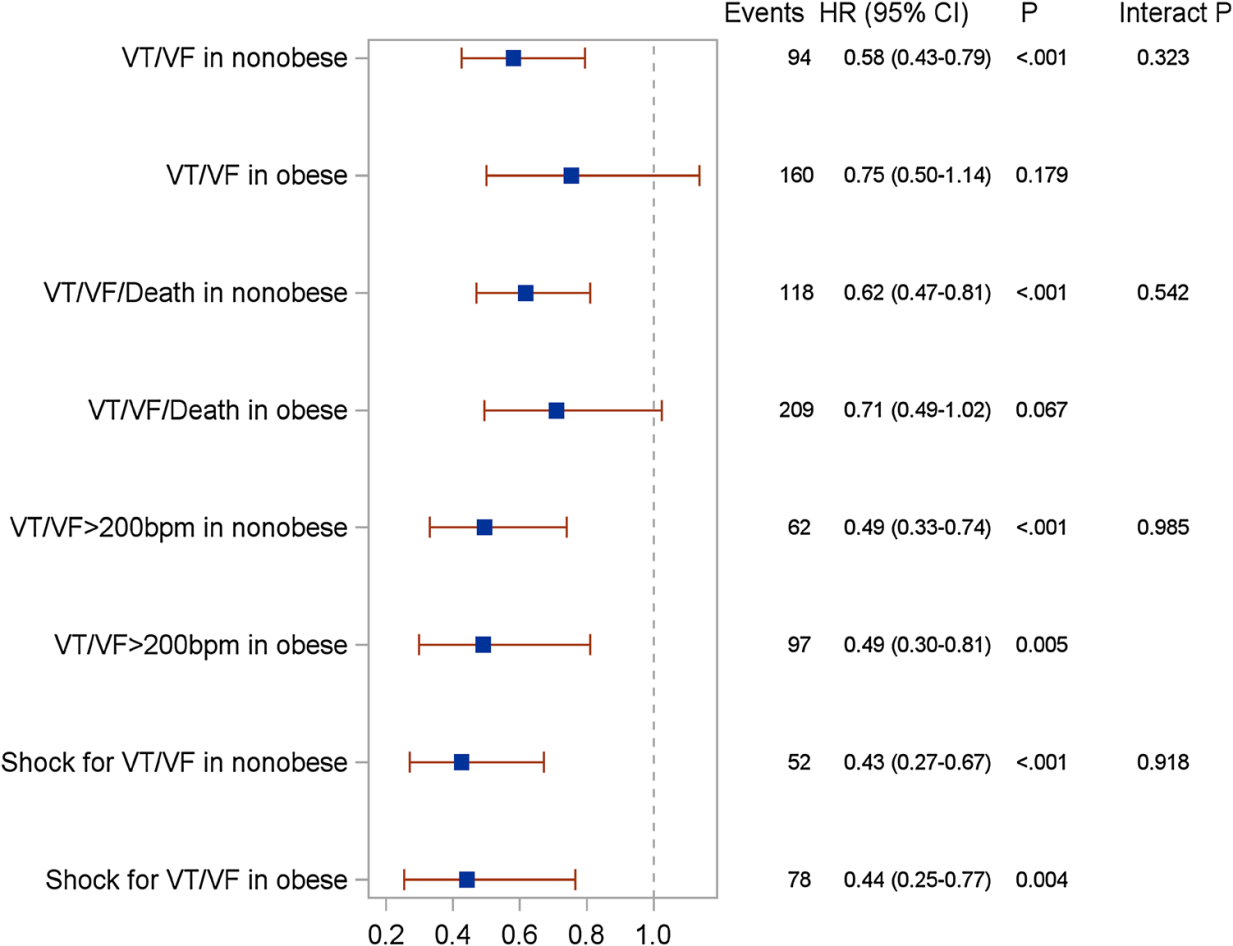
The Effect of CRT-D vs. ICD in obese and non-obese patients on the risk of appropriate implantable cardioverter—defibrillator therapy. (VT/VF-ventricular tachycardia/ventricular fibrillation)

However, multivariable analysis showed that CRT-D treatment significantly reduced the risk of appropriate ICD therapy in non-obese patients ([HR]; 0.58 [CI]: 0.42–0.79; p < 0.001) and to a lesser degree in obese patients (HR 0.75, 95 % CI 0.5–1.38; p = 0.179), however interaction p value was not significant p = 0.323. Significant reduction in risk of fast VT/VF was observed in non-obese ([HR]; 0.49 [CI]: 0.33–0.73; p < 0.001) and obese patients ([HR]; 0.49 [CI]: 0.29–0.81; p < 0.01) (Fig. 3).

### The effect of CRT-D on the risk of recurrent appropriate implantable cardioverter—defibrillator therapy and death

**Fig. 4:**
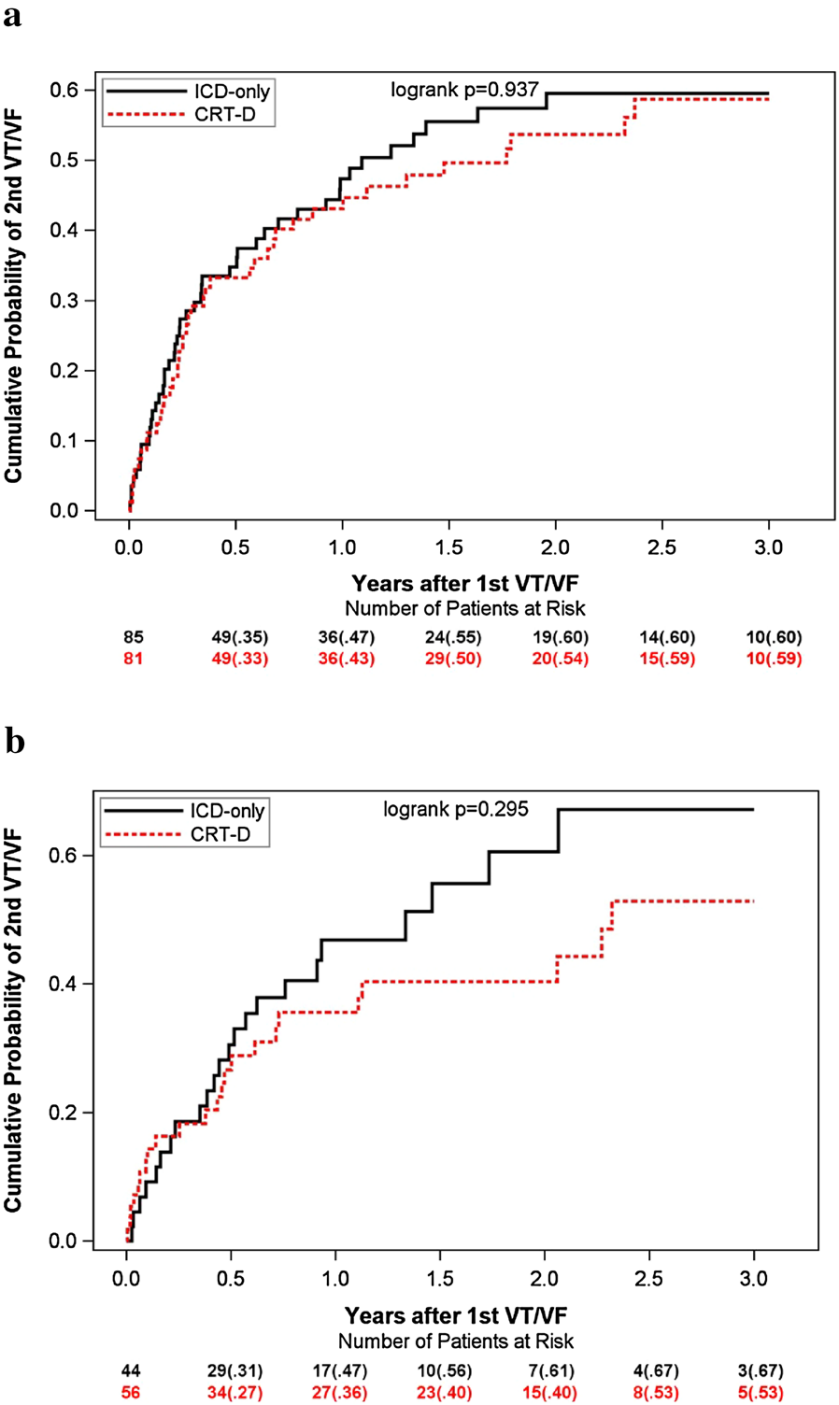
Cumulative probability of VT/VF following a first VT/VF event by treatment arm in: **a** non-obese and **b** obese patients

Among patients who experienced a first incidence of VT/VF, Kaplan–Meier survival analysis showed no statistically significant difference between treatment groups by obesity status with regards to the occurrence of a second VT/VF (Fig. 4). Consistently, multivariable model showed that the benefit of CRT to reduce the VT/VF end point was not evident in non-obese and obese patients among those who experienced a first event (Table 4). CRT-D therapy was effective in reducing VT/VF faster than 200 bpm (p = 0.086) and VT/VF episodes requiring ICD shocks (p = 0.031) in obese patients only.

**Table 4:** The effect of CRT-D in obese and non-obese patients on the risk of subsequent appropriate ICD therapy or death

	Number of events	Non-obese	Number of events	Obese	p value
		Adjusted HR (95 % CI) p value		Adjusted HR (95 % CI) p value	
Subsequent VT/VF					
VT/VF	371	1.05 (0.79–1.39) 0.748	272	1.05 (0.73–1.52) 0.797	0.508
VT/VF greater than 200 bpm	85	0.910 (0.52–1.59) 0.741	68	0.59 (0.32–1.01) 0.086	0.243
Shock delivered for VT/VF	82	0.81 (0.42–1.55) 0.522	55	0.45 (0.22–0.93) 0.031	0.135
Death					
VT/VF	70	2.02 (1.14–3.57) 0.014	36	2.79 (1.37–5.68) 0.004	0.477
VT/VF greater than 200 bpm	70	2.58 (1.33–4.98) 0.004	36	2.39 (1.07–5.38) 0.033	0.885
Shock delivered for VT/VF	70	3.15 (1.6–6.2) <0.001	36	2.21 (0.94–5.12) 0.064	0.514

After adjustment for race (Black/African American), age at enrollment, creatinine ≥1.4, female sex, left ventricle end diastolic volume index, myocardial infarction prior to enrollment, enrollment NYHA classification, prior hospitalization during prior year, QRS <150, ventricular arrhythmias requiring treatment prior to enrolment

Among non-obese patients who had an appropriate ICD therapy a higher risk of death was observed ([HR]; 2.02 [CI]: 1.14–3.57; p = 0.014), similarly to obese patients ([HR]; 2.79 [CI]: 1.37–5.68; p = 004) (Table 4) comparing to those who did not have appropriate ICD therapy.

## Discussion

To our knowledge, the present study is the first to assess the effect of obesity on the risk of VT/VF and recurrent VT/VF in response to CRT-D treatment. In our current analysis obesity was not associated with a higher risk of first and subsequent ventricular tachyarrhythmias and did not diminish clinical benefit of cardiac resynchronization therapy to reduce risk for appropriate therapy delivered for VT/VF. Consistently both non-obese and obese patients showed higher risk for death after the occurrence of the appropriate ICD therapy.

Our previous MADIT II study reported a higher rate of VT/VF in obese post-infarction patients [17]. MADIT II was a post-infarction population study without a requirement of heart failure. The overall rate for VT/VF was 10 % higher than in MADIT-CRT. The MADIT CRT population consisted of patients with both ischemic and non-ischemic cardiomyopathy [24]. Additionally, pharmacological treatment and prevention strategies for heart failure optimize over the years and this may contribute to lower rate for VT/VF in current population [25]. Success strategies for ICD implantation are also similar in obese and non-obese [26]. CRT-D reduces the risk for heart failure, death and VT/VF [20, 27, 28], but significant evidence suggests that the benefit derived from CRT varies by baseline conduction disturbances therefore our study included only patients with LBBB [21].

The mechanism of cardiac arrhythmias in obesity includes: heart remodeling including left ventricle hypertrophy, subclinical systolic impairment and diastolic dysfunction, pericardial adipose tissue inflammatory cytokine secretion and sympathetic over-activity [11]. Pathological myocardial changes such as myocyte hypertrophy, fibrosis, focal myocardial disarray, fatty infiltration and increased epicardial fat may contribute to increased risk of arrhythmias [7]. Despite the strong evidence that obesity may predispose to ventricular arrhythmias our current study did not support prior observations. In patients with obesity and heart failure, association between body mass index and subsequent cardiovascular risk is complex and known as “obesity paradox” [29, 30], suggesting that role of obesity accompanied establish HF, the effect on clinical outcome including risk for VT/VF may be neutral or even positive at this stage of disease [31].

In summary, strategies to reduce risk for negative health outcomes in people with obesity should be implemented early before the development of HF. This is supported in our analysis because obese patients are 5 years younger than people without obesity. The preventive strategies may include adequate body weight monitoring which may translate into prevention of HF development and consecutive ICD or CRT therapy [19].

Limitation of our study relates to a retrospective and nonrandomized nature of this post hoc analysis. An adjusted multivariate analysis was performed, taking into account many confounders associated with analyzed end point and those that played a significant role on this outcome in our population.

In conclusion, our findings indicate that obesity in mild heart failure did not diminish clinical benefit of cardiac resynchronization therapy. Patients with obesity should be offered CRT-D treatment and obesity should not preclude the use of CRT-D treatment when clinically indicated.

## Additional files

10.1186/s12933-016-0401-xThe risk of appropriate implantable cardioverter—defibrillator therapy delivered for VTVF a full multivariable model.
10.1186/s12933-016-0401-xAssociation between appropriate ICD therapy delivered for VT/VF and the BMI level.
