# Supplementary material for: Effect of obesity on the effectiveness of cardiac resynchronization to reduce the risk of first and recurrent ventricular tachyarrhythmia events
Source: Cardiovasc Diabetol. 2016 Jul 7;15:93. doi: 10.1186/s12933-016-0401-x (PMC4936234; doi:10.1186/s12933-016-0401-x)
Supplement: Supplementary file 1 — 10.1186/s12933-016-0401-xThe risk of appropriate implantable cardioverter—defibrillator therapy delivered for VTVF a full multivariable model. [file 12933_2016_401_MOESM1_ESM.docx]

**Table 1 Supplementary. The Risk of Appropriate Implantable Cardioverter – Defibrillator Therapy delivered for VTVF a full multivariable model.**

| Number of Observations Read 1264  Number of Observations Used 1178  254 total Events | | | |
| --- | --- | --- | --- |
| **Parameters** | **HR** | **95% CI** | **P value** |
| Obesity | 1.05 | 0.81- 1.37 | 0.703 |
| The effect of CRT vs. ICD | 0.58 | 0.43 - 0.79 | <0.001 |
| Black/African American | 1.97 | 1.31 - 2.97 | 0.001 |
| age at enrollment, | 0.98 | 0.97 - 0.99 | 0.004 |
| creatinine >= 1.4 | 0.64 | 0.45 - 0.90 | 0.012 |
| female | 0.57 | 0.42 - 0.79 | 0.001 |
| left ventricle end diastolic volume index | 1.01 | 1.01 - 1.01 | <.001 |
| myocardial infarction prior to enrollment | 1.68 | 1.25- 2.25 | 0.001 |
| enrollment NYHA classification | 1.75 | 1.10 - 2.78 | 0.018 |
| prior hospitalization during prior year | 0.74 | 0.57 - 0.96 | 0.022 |
| QRS < 150 | 1.62 | 1.23 - 2.15 | 0.001 |
| ventricular arrhythmias requiring treatment prior to enrolment | 2.39 | 1.65- 3.44 | <.001 |
