# Supplementary material for: Effect of obesity on the effectiveness of cardiac resynchronization to reduce the risk of first and recurrent ventricular tachyarrhythmia events
Source: Cardiovasc Diabetol. 2016 Jul 7;15:93. doi: 10.1186/s12933-016-0401-x (PMC4936234; doi:10.1186/s12933-016-0401-x)

Figure 1 supplementary: Association between appropriate ICD therapy delivered for VT/VF and the BMI level.


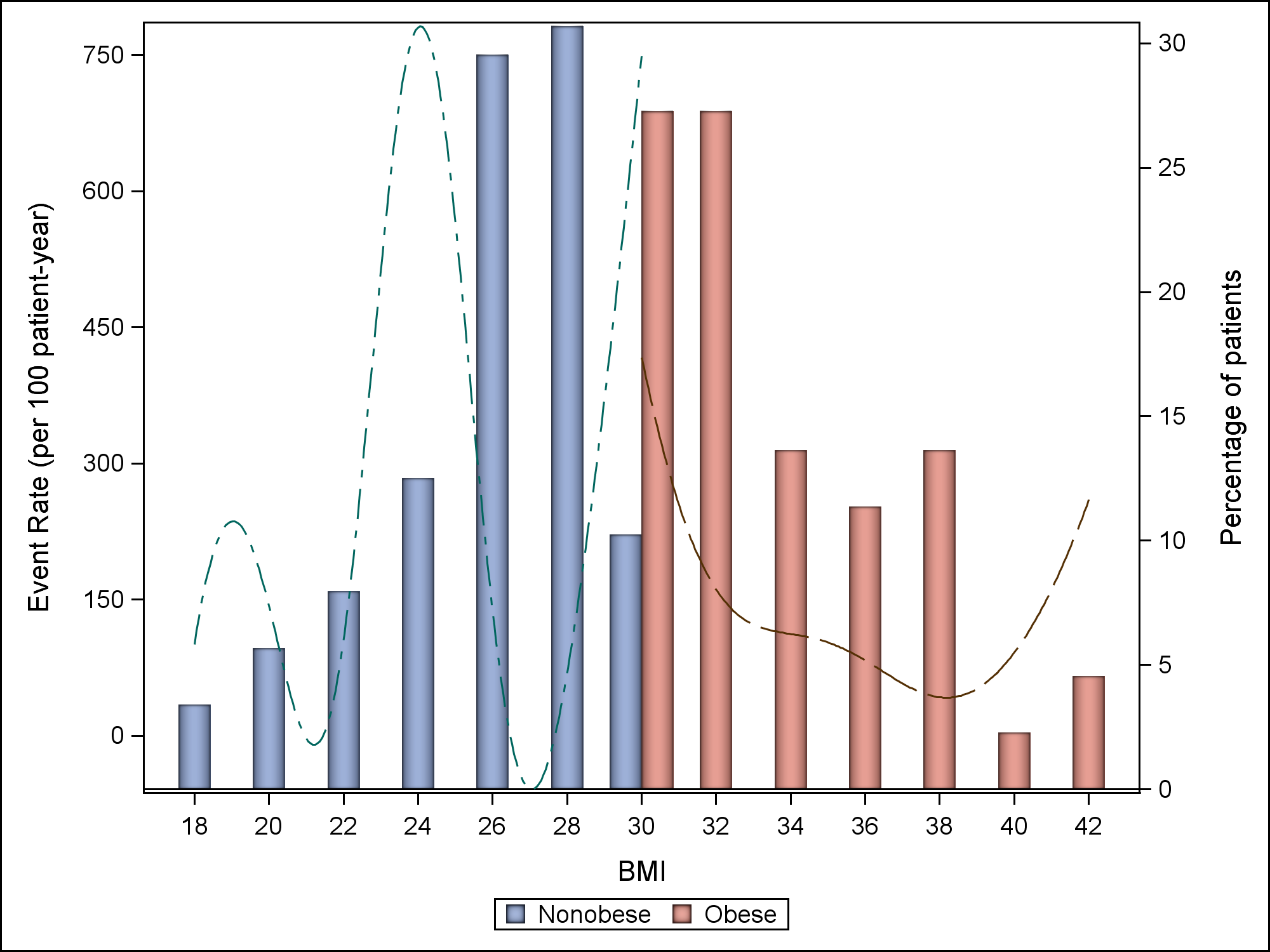

Supplement: Supplementary file 2 — 10.1186/s12933-016-0401-xAssociation between appropriate ICD therapy delivered for VT/VF and the BMI level. [file 12933_2016_401_MOESM2_ESM.docx]
